# Life Cycle and Suicidal Behavior among Women

**DOI:** 10.1155/2013/485851

**Published:** 2013-02-28

**Authors:** Pablo Mendez-Bustos, Jorge Lopez-Castroman, Enrique Baca-García, Antonio Ceverino

**Affiliations:** ^1^Department of Psychology, Catholic University of Maule, Avenida San Miguel 3605, Talca, Chile; ^2^Department of Psychiatry, Fundacion Jimenez Diaz Hospital, Autonoma University, CIBERSAM Avenida Reyes Catolicos 2, 28040 Madrid, Spain; ^3^Department of Psychiatry, New York State Psychiatric Institute Columbia University, 1051 Riverside Drive, New York, NY 10032, USA; ^4^Centro de Salud Mental de Hortaleza, 28033 Madrid, Spain

## Abstract

It is nowadays accepted that, independently of methodological issues, women commit fewer suicides than men but make more frequent attempts. Yet, female suicidal risk varies greatly along the lifetime and is linked to the most significant moments in it. A wide analysis of the existing literature was performed to provide a narrative description on the evolution of female suicidal rates from childhood to old age, considering the milestones in their life history. A detailed analysis of gender differences in suicidal behavior is key to establish preventive measures and priorities. More specific studies are needed to adapt future interventions on female suicide.

## 1. Introduction

A vast majority of epidemiological studies performed in diverse cultures and countries show gender differences in suicidal behavior. In developed countries, the completed suicides are 2 to 4-fold more frequent among men [1–3], while suicide attempts are 2 to 3-fold more frequent among women [4–6]. However, suicide rates vary significantly between regions and countries. In Europe, northern countries report higher suicide rates [7]. Developed countries have higher male to female ratios than Asian countries [8, 9], although the estimated global male/female suicide ratio is 1.67 to 1 and not 3 to 1 [10]. Young women may be particularly exposed to suicidal risk [6, 11]. For instance, during 2005 suicide was the fourth cause of death in the United States (US) among women aged 15–44 years [12]. The rates of suicidal ideation and attempts among females are notably increased after puberty [13]. It has been calculated that in the US a woman attempts suicide every 78 seconds and dies of it every 90 minutes [14]. 

The higher frequency of completed suicides among men and suicide attempts among women is called the gender paradox and has been reported on many different countries. This paradox is absent in India and China where women and men present similar suicide rates [10, 15] due to the high rates of completed suicide among rural young women [16, 17]. In addition, suicide among Indian and Chinese women may be favored by the use of lethal methods such as self-burning in India and pesticides in China [17–19]. It is of note that female suicide rates in South Korea have increased from 1.1 (1986) to 4.2 (2005) per 100.000 [20]. The limitations to obtain national suicide data from undeveloped countries remark the presumed importance of cultural dimensions. It must be remembered that WHO counts with trustworthy information on death causes covering about 13% of the world population and actualized mortality data on 25% of the world population is lacking [21].

Not considered to be a methodological artifact [4], a lack of agreement on the origin of the gender paradox persists. Proposed explanations are based on the differential suicidal methods, which may condition lethality, disposal, and cultural acceptance [3, 22]. Usually, males use methods such as shooting by firearm, hanging, or suffocation, while females attempt poisoning, wrist cutting, or falling from heights [2]. Durkheim [23] suggested that suicide is influenced by individual traits but also by the characteristics and changes of the society. Males appear to be more affected by external factors, such as economic crisis, than females [24–27].

The study of sexual dimorphism is constraint by the variations of risk factors between generations. For example, McIntosh [28] analyzed the rates of suicide between baby-boomers (born in the 1943–1960 period) and the 13th generation (born in the 1961–1981 period) in the US. Following his results, the suicidal risk is increased among subjects of the 13th generation when considering the same chronological age. Some authors have informed as well on a reduction in the global rates of suicide in recent years, especially among aged women and despite a subtle increase among young men [29]. The WHO/EURO multicentre study [30] reported that suicide rates had diminished by 17% among men and 14% among women from 1989 to 1992. Others point out that the gender rate of completed suicide in the USA has remained stable around 2.5 : 1 (2.5 fold more frequent completed suicides among men) from 1930 to 1971, but has increased ever since reaching a proportion of 4.4 : 1 in the last decade of the nineties [14]. Nevertheless, studies analyzing different time periods in developed countries found that the suicide rate in women has increased over time [31, 32]. 

Besides, global ratios may conceal bigger differences among gender during the vital cycle. Hawton and Harriss [33] analyzed a large sample of self-aggressions admitted to the hospital in a 10-year interval. Gender ratio was globally close to 1.5 women for each man. However, this coefficient varied greatly between age groups and decreased with advancing age, from 8:1 among the younger (10–14 years of age) to 0.8:1 among the elder (>50 years of age). 

**Figure 1:**
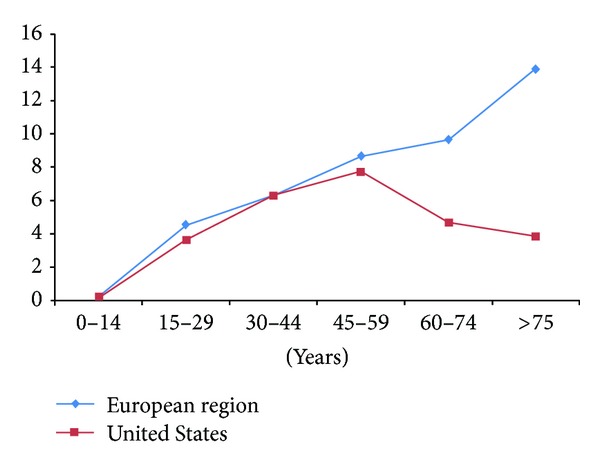
Evolution of female suicide rates in Europe and the United States 1999–2010 (Source: http://data.euro.who.int/hfamdb/, http://webappa.cdc.gov/sasweb/ncipc/mortrate10_us.html)

The present study is focused on the longitudinal evolution of the suicidal behavior in women and particularly at some significant moments of women's lifetime: childhood/adolescence, reproductive cycle/pregnancy, middle-aged/marital status, and old age (see Figure 1 and Table 1). 

## 2. Suicidal Behavior in Childhood, Adolescence, and Youth

Infantile suicide is an unusual occurrence. However, the number of suicides among children and adolescents till 14 years of age appears to be increasing in several countries. Rates are varying from 0 to 3.1/100000 between countries with an estimated 0.6/100000 global rate and a 2 : 1 male/female coefficient [34]. These rates rise towards adolescence due to a greater planning and more lethal suicide attempts, and together with a higher prevalence of mental disorders and substance abuse [35, 36]. 

Some studies observed the gender paradox among subjects aged 10–19 years. Adolescent women from 13 years of age show an abrupt increase of suicidal ideation, plans, and attempts [37]. The rates of suicidal ideation and attempts are consistently increased after puberty among females when compared to male adolescents [13, 38]. The highest rates of suicide attempts [39] or parasuicides [40] appear earlier in adolescent women compared to men, with a time gap of about 3 years [39]. In addition, teenage girls that committed suicide more often had previous attempts and conflicts with their parents and left a note than male groups [41, 42]. Suicidal behavior among female adolescents should be a worrying problem for institutions and researchers [43].

Suicide is the third leading cause of death for persons aged 10–14 years and 15–24 years, the second leading cause for persons aged 25–34 years in the US [2], and the second cause of mortality after accidental deaths in Europe [44]. According to a recent study on 14738 suicides committed in 15 European countries among youths aged 15–24 years, men had a 3.7-fold higher risk of completed suicide than women [3]. Some authors have suggested that this difference between male and female adolescents is due to methodological issues, such as an overrepresentation of male subjects in the group of suicides aged 15–19 years [45]. However, gender differences could be explained by greater levels of aggression, more frequent substance use disorders, and more lethal methods in males than females [35, 46]. This situation changes when considering Asian countries, for instance in Hong Kong the suicide rates (per 100000) among persons aged 15–19 were 5.1 for males and 5.2 for females [47]. Gunnell et al. [48] showed that although the suicide rate in England and Wales is still higher in men, the difference in recent years has decreased compared to women (1950–1998). Suicide is also the world leading cause of death in women aged 15–24 years, mainly in low-income and middle-income countries, according to Patton et al. [49]. In addition, suicides by hanging/suffocation may be augmenting among U.S. women aged 15–34 years [50] and 10–19 years [51]. Åsgård et al. [52] analyzed the causes of death in Sweden from 1952 to 1981, finding a tendency towards lower ages and higher female suicidal risk along this period. 

Brent et al. [35] have compared several studies on psychological autopsies among adolescents with their own sample. They found that females used generally less lethal means, such as self-poisoning by overdose, with a more frequent prevalence of affective disorders and previous attempts. However, adolescent women who commit suicide may use increasingly violent methods such as shooting by firearms or hanging [41]. Klein et al. [53] found that the method most commonly used in the 10–19 years group was jumping form a height, while the 20–49 age group used other methods such as poisoning, hanging, strangulation, suffocation, or drowning. Other studies in western populations found that the most common suicidal methods in women aged 10–24 years were hanging/suffocation, drug poisoning, and jumping [3, 54]. Thus, if women used more lethal means the gender difference in suicide rates could be reduced [55]. However, the highest suicidal risk among female adolescents precede 2-3 years that of male adolescents, and by 19 years of age the risk is similar [43]. Saunders and Hawton [56] suggested that the initiation of menarche is the moment when gender differences in the ratios of affective disorders and suicide behavior move apart.

## 3. Role of Reproductive Cycle and Maternity

Consistent evidence of an association between menstrual phase and completed suicide has not been found [56]. Non-fatal suicidal behavior and suicidal ideation seem to be more frequent when estrogen levels are lowest during the menstrual cycle, in particular the late luteal and follicular phases [56–58]. Besides, suicide attempters have shown higher prevalence of premenstrual symptoms and premenstrual dysphoric disorder than the general population [57].

Several studies seem to confirm that maternity plays a more important role than marriage in the decreased risk for completed suicide among middle-aged women when compared to men. Actually, mothers having more children show an enhanced protection [59]. Being pregnant [33] and having a child of less than two years of age [60] have also been associated with lower suicidal risk. Moreover, as the age of the youngest child diminishes, suicide risk is reduced to a greater extent [59]. Women living with a partner and children that changed to living with only a partner were overrepresented among parasuicidal women in the WHO study [30]. 

Different authors support the idea that the birth of a child is a protective factor against fatal and non-fatal self-harm, especially in the first year after delivery [59, 61, 62]. However, this protective function differs in pregnant women with psychiatric disorders. Between 10–25% of pregnant and postpartum women experience depressive disorders [63, 64] or anxiety disorders [65, 66]. These women are more likely to complete suicide, especially within the two first months of the postpartum [67, 68]. In addition, pregnant teens represent a high risk group, with an estimated 16–44% prevalence rate of depression [69, 70]. Teen mothers are more likely to present suicidal thoughts, or attempts especially if it is the first pregnancy or if the pregnancy is unplanned [71–74]. 

Suicide is the fourth cause of maternal deaths in the world [75] and the leading cause of death in first-year postpartum women in the United Kingdom [76, 77]. The risk of suicide was calculated to be 70 times higher in women with psychiatric disorders during the first year after childbirth compared to the general female population [78]. In the same vein, Gissler et al. [68] reported a suicide rate of 11 per 100.000 in a large sample of Finnish postpartum women. In this study, suicide rates associated with childbirth were close to half of those among non-pregnant women aged 15–49 years, but adolescent mothers were three times more likely to commit suicide than other females in their age group.

Suicidal behavior and suicide rates may be increased after an abortion, particularly when induced [79, 80]. In fact, induced abortion may increase suicidal risk in relation with the impact of the decision itself, because prior to the abortion no difference in suicidal risk was found with women completing their pregnancy [81]. However, findings on mental health consequences of abortion have been contested, and the recent literature review limited the validity of studies to date [82]. 

Miscarriage has also been linked to an increased maternal suicide risk [68, 81]. Other factors associated with an increased risk of suicide in pregnant women and after childbirth were single, unmarried, or divorced marital status, low income, having thoughts about abortion, unemployment, occupational instability, and poor social support [65, 83–85]. Finally, another dimension associated with female suicide but less studied is infertility. Kjaer et al. [86] found in a sample of 51221 Danish women, that those who succeeded in the treatment of infertility had half the risk of suicide than the unsuccessful ones. 

## 4. Suicide in Middle-Aged Women, Marriage, and Divorce

In the US, female suicide is concentrated in the 35–64 years age group (64.8%), with a 9.1/100000 peak between those aged 45–54 years [2]. Similar results have been reported for England and Wales [25]. Societal changes lead many women in this age group to become economically active, maybe increasing the risk of suicide among them [48, 93] as well as the mental health problems [87]. From 50 years of age, the suicide rates among women tend to diminish progressively [4, 94] till old age, when rates start increasing again (Figure 1). White and Holmes [89] found that suicide rate in women increases with age reaching its peak at 35–44 years. Yet, depression and suicide ideation have been associated with the perimenopause phase in women when compared to premenopausal and postmenopausal [95]. 

According to the exist literature, married women are less prone to suicide than single, divorced, and widowed women [88]. Never-married, divorced, or widowed women conduct most suicides (60.4%) in the US [2]. Cutright et al. [88] analyzed retrospective data from 12 developed countries to explain the differences in suicide between married and non-married women. They concluded that the compatibility of marital status with the corresponding age group was the best explanation of these differences, but the results were limited by not considering the influence of maternity. Divorce affects in a singular way the risk of suicide among women. They present lower suicide rates after divorce than men, but the gender protection seems to decrease with advancing age [96].

## 5. Suicidal Behavior among the Oldest Women: Death of Partner or Child

Advanced age seems to increase the divergence between sexes in the rate of completed suicide among the elder [22]. Whereas suicide attempt rates diminish with age independently of gender [97], the rates of completed suicide augment with age [92]. This increase is particularly prominent among men [53] reaching 6–12 times higher rates than women in western countries [98]. This important difference has been attributed to a better planning, fewer warnings of suicidal intent, and the use of more lethal methods, mainly firearms and hanging/suffocation [91, 98–100]. However, the male to female suicide ratio did not change in Eastern Europe or South America in the group aged over 65 years [101], and it even decreased in the US [6]. 

**Figure 2:**
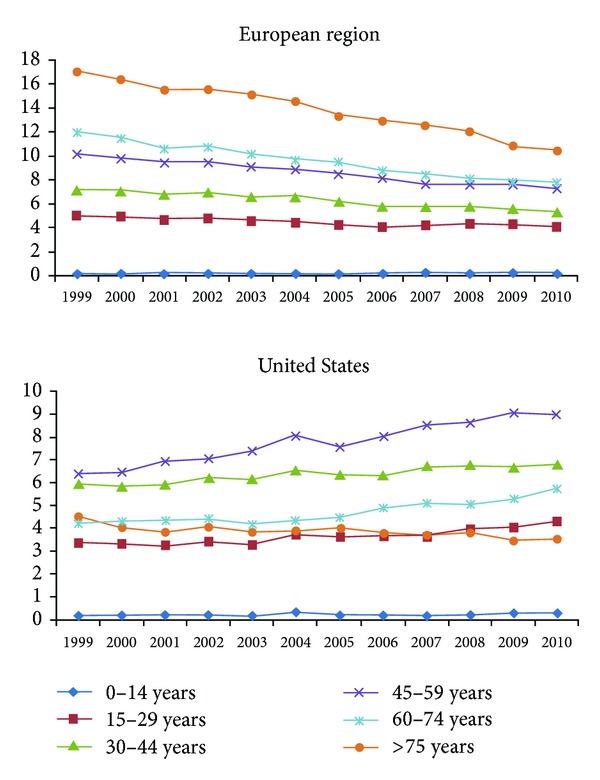
Comparison of longitudinal trends in female suicide rates between Europe and the United States 1999–2010. Source: http://data.euro.who.int/hfamdb/, http://webappa.cdc.gov/sasweb/ncipc/dataRestriction_inj.html.

Female suicide rates in western countries increase with advancing age until they reach a peak around menopause. However, the evolution of suicide rates among older women may vary greatly depending on the country. In Europe they appear to continue their growth at a lower pace but sustainable increased in the oldest age groups (Figure 1) [53, 102], and similar results have been reported in Russia [27], Korea [103], and China [104]. The Centers for Disease Control and Prevention (CDC) report a declining trend in the US, 5.8 for those aged 60–69 years, 4.2 for those aged 70–79 years, and 2.7 for those aged over 80 years [2]. The largest differences between Europe and the US with regards to female suicide rates are seen in this group of age (Figure 1), although longitudinal trends show an approximation in the rates of both regions in recent years (Figure 2).

Widowed, divorced, and never married old women are at greater risk of completing suicide [91, 92]. The death of the partner occupies a prominent place to explain the high rates of suicide they present. Following the work by Erlangsen et al. [90] old persons present a 15-fold higher risk of suicide after the loss of their partner than middle-aged persons. Though women are also affected, suicide rates and time needed for recovery are particularly increased among men. In an interesting study by Agerbo [105], conjugal bereavement was found to raise spousal suicide risk, and especially when death had been the result of a suicide. He also found that suicide risk when other causes produced the death of the partner was three-fold higher among men than among their feminine counterpart. Some studies found that the main methods used to completed suicide by this age group are poisoning and firearms [53, 98]. 

Parental suicide risk is highly influenced by the loss of a child [59]. Risk is enhanced in the first month after the death, with younger age of the child or if result of a suicide [59]. The increased risk of suicide is independent of gender, but having another child constitutes a protective factor for the mother [105]. 

## 6. Conclusions 

Suicidal behavior presents important differences between men and women. Men are more prone to completed suicide but women have more frequent suicide attempts. It is nowadays accepted that this fact is independent of methodological issues. Several hypotheses have been proposed to explain this difference; some of them underline biological aspects, while others focus mainly on the feminine role and psychosocial aspects of gender. In any case, gender is one of the most frequently replicated predictors of suicide, and a detailed analysis of gender differences in suicidal behavior is important to establish preventive measures and priorities. Besides, suicide risk is not regular along the female lifecycle and the literature revision revealed large cross-national differences. Intervention on suicide must therefore be adapted specifically to the different populations. Studies analyzing the evolution of suicidal behavior in women and associated factors in the most significant milestones of their life history are needed. 

The evolution of female suicide rates in Europe and the United States diverges in the 1999–2010 period. Suicide rates increase in the old age among European women, while an opposite trend for that age period is seen in the U.S. (Figure 1). Consequently, the longitudinal trends show the highest suicide rates in European women over 75 years, while the highest rates in the US correspond to women of 45–59 years of age (Figure 2). These differences demonstrate the importance of cultural and sociodemographic variables in the analysis and should be considered for the development and implementation of prevention programs. 

## Figures and Tables

**Table 1:** Female suicide across the life cycle: main studies.

Authors	Population	Main results
Childhood, adolescence, and youth women		

Biddle et al., [50]	England and Wales, 1968–2005. Men and women aged 15–34 years	Suicide rates stability over time. In the 21st century recording lowest rate
Grøholt et al., [41]	Norway, 1990–1992. All suicide in people under 20 years	Teenage girls suicide victims died mainly by hanging. They were more often affected by problems with parents, wrote farewell note, and had previous suicide attempts
Eaton et al., [37]	Youth Risk Behavior Surveillance System (YRBSS) USA, 2007. 1268 primary students	Adolescent women from 13 years of age show an abrupt increase of suicidal ideation, plans, and attempts
Gunnell et al., [48]	Mortality data England and Wales, 1950–1998	Suicide rates decreases in women aged over 45 years. Risk greater in women aged 25–34 years associated to participation in the workforce
Lewinsohn et al., [43]	USA (Western Oregon), 1987–1989. 1709 adolescents (aged 14–18)	Suicide attempts hazard rate in female adolescents greater than males adolescents. Adolescent suicidal behavior predicted suicide attempts during young adulthood for females

Role of reproductive cycle and maternity		

Appleby, [61]	England and Wales, 1973–1984. Women aged 15–44 years committed suicide in the year after childbirth or during pregnancy	Women in the first year after childbirth or during pregnancy have a low-risk suicide despite their high rate of psychiatry morbidity women who committed suicide after childbirth most often did at the first month
Czeizel, [71]	Budapest, 1960–1993. 1044 pregnant women aged 14–44 years	Maximum number of suicide attempts in pregnant women occurs in the group from 18 to 20 years. Most unplanned pregnancies and main method used poisoning
Da silva et al., [65]	Brazil, 2006–2008. 1414 women pregnant treated in the public health system	There is greater suicidality in pregnant women who have depressive and anxiety symptoms
Gissler et al., [68]	Finland, 1987–1994. 1347 women aged 15–49 years that committed suicide	The risk of suicide was at its highest during the first two months after the end of pregnancy and mainly in the age group 35–39 years
Samandari et al., [85]	North Carolina surveillance and vital statistics data from 2004–2006. Women reproductive age, 14–44 years	Greater percentages of pregnant/postpartum suicide victims never married compared to no pregnant/no postpartum suicide victims

Middle-aged women		

Burrows et al., [31]	Canada (Québec), 1990–2005. People 10 years and older	Suicide mortality in women increases in the time. Rate suicide is highest between 25 and 44 years (2002–2005)
Bramness et al., [87]	Norwegian, 1994–2007. 131362 people (69774 women) aged 39–44 years	More self-report mental health problems among females than males. Increased risk of suicide with higher self-report depressive and anxiety symptoms
Cutright et al., [88]	Suicide rates of married and not married females in 12 developed countries, 1960	The suicide rate is higher in not married females mainly age group 35–44 years. Being married is a protective factor.
Karch et al., [2]	United States, 2009. National Violent Death Reporting System (NVDRS). 15981 fatal incidents (60.6% suicides)	Females among ages 35 and 64 years accounted for 64.8% of suicides. Rates suicide for females peaked at 9.1 per 100,000 among those aged 45–54 years
White and Holmes, [89]	Mortality database WHO, men and women aged 15–44 years across 44 countries	Suicide rate in women increases with age. Group 15–24 years (14.1), group 25–34 years (21.7), and group 35–44 years (23.8)

Oldest women		

Erlangsen et al., [90]	Danish population, 1994–1998. People 50 years or above	During first year of widowhood the suicide risk increases in ages over 80 years. The highest rate of suicide is reached in the group 65–79 years and then declines over 80 years
Klein et al., [53]	Switzerland (Canton), 1995–2007. 3431 cases of suicide	Suicide risk increases with age. Women's group of the 50–89 years rates the highest. Main methods used poisoning, hanging, and firearm
Pridemore and Spivak, [27]	Mortality date Russian, 1965–1999	Suicide rates increased about 17% in the last three decades. Suicide rate in women increases with age reaching its peak over 80 years. Risk factor in females that reside alone
Wanta et al., [91]	Wisconsin's. USA, 2001–2006. People over 65 years	The suicide rates in women decrease with age. Protective factor is being married.
Zeppegno et al., [92]	Date on suicide in Italy (Novara and Verbania), 1990–2000. People 65 and older years	Suicide rates in women increase over 74 years. Greater risk of suicide in divorced, widowed, and single
